# Phytochemicals as Novel Therapeutic Strategies for NLRP3 Inflammasome-Related Neurological, Metabolic, and Inflammatory Diseases

**DOI:** 10.3390/ijms20122876

**Published:** 2019-06-13

**Authors:** Carolina Pellegrini, Matteo Fornai, Luca Antonioli, Corrado Blandizzi, Vincenzo Calderone

**Affiliations:** 1 Department of Pharmacy, University of Pisa, 56126 Pisa, Italy; vincenzo.calderone@unipi.it; 2 Department of Clinical and Experimental Medicine, University of Pisa, 56126 Pisa, Italy; mfornai74@gmail.com (M.F.); lucaant@gmail.com (L.A.); c.blandizzi@gmail.com (C.B.)

**Keywords:** NLRP3 inflammasome, neurological diseases, psychiatric diseases, metabolic diseases, inflammatory diseases, phytochemicals

## Abstract

Several lines of evidence point out the relevance of nucleotide-binding oligomerization domain leucine-rich repeat and pyrin domain-containing protein 3 (NLRP3) inflammasome as a pivotal player in the pathophysiology of several neurological and psychiatric diseases (i.e., Parkinson’s disease (PD), Alzheimer’s disease (AD), multiple sclerosis (MS), amyotrophic lateral sclerosis, and major depressive disorder), metabolic disorders (i.e., obesity and type 2 diabetes) and chronic inflammatory diseases (i.e., intestinal inflammation, arthritis, and gout). Intensive research efforts are being made to achieve an integrated view about the pathophysiological role of NLRP3 inflammasome pathways in such disorders. Evidence is also emerging that the pharmacological modulation of NLRP3 inflammasome by phytochemicals could represent a promising molecular target for the therapeutic management of neurological, psychiatric, metabolic, and inflammatory diseases. The present review article has been intended to provide an integrated and critical overview of the available clinical and experimental evidence about the role of NLRP3 inflammasome in the pathophysiology of neurological, psychiatric, metabolic, and inflammatory diseases, including PD, AD, MS, depression, obesity, type 2 diabetes, arthritis, and intestinal inflammation. Special attention has been paid to highlight and critically discuss current scientific evidence on the effects of phytochemicals on NLRP3 inflammasome pathways and their potential in counteracting central neuroinflammation, metabolic alterations, and immune/inflammatory responses in such diseases.

## 1. Introduction

A growing body of evidence highlights the relevance of nucleotide-binding oligomerization domain leucine-rich repeat and pyrin domain-containing protein 3 (NLRP3) inflammasome in the pathophysiology of several autoinflammatory syndromes (i.e., cryopyrin-associated autoinflammatory syndromes (CAPS)), neurological and psychiatric diseases (i.e., Parkinson’s disease (PD), Alzheimer’s disease (AD), multiple sclerosis (MS), amyotrophic lateral sclerosis (ALS), and major depressive disorder (MDD)), metabolic disorders (i.e., obesity and type 2 diabetes), and chronic inflammatory diseases (i.e., arthritis and intestinal inflammation) [1,2,3,4]. In particular, the NLRP3 inflammasome complex, including NLRP3, adaptor protein apoptosis-associated speck-like protein (ASC), and pro-caspase-1, through the processing and release of interleukin (IL)-1β and IL-18, acts as a key player both in coordinating the host physiology and shaping the central and/or peripheral immune/inflammatory responses in neurological, metabolic, and inflammatory diseases [5]. Indeed, an overactivation of NLRP3 inflammasome signaling has been observed in the brain and blood of patients with neurological disorders, adipose tissue macrophages from obese and diabetic patients, as well as patients with rheumatoid arthritis (RA) and inflammatory bowel diseases (IBDs) [2,6,7,8,9]. In keeping with this knowledge, it is becoming increasingly appreciated that drugs targeting the NLRP3 pathway could represent suitable therapeutic options for the management of a large variety of diseases [10]. For instance, the pharmacological blockade of NLRP3 signaling has been found to exert beneficial effects in animal models of PD, MS, obesity, type 2 diabetes, arthritis, and colitis [10]. Of note, given the great interest in the therapeutic potential of phytochemicals, in terms of prevention, cure, and maintenance of remission, intensive efforts are being made to characterize the effects of natural compounds targeting NLRP3 pathways in neurological, metabolic, and inflammatory diseases [11]. In this regard, several studies have shown that various phytochemicals, including polyphenols and glucosinolates, counteract neuroinflammation, metabolic alterations, and immune/inflammatory responses in experimental models of diseases via inhibition of NLRP3 signaling [12,13,14].

Based on the above background, the present review article intents to provide an integrated and critical overview of the available clinical and experimental evidence about the role of NLRP3 inflammasome in the pathophysiology of neurological, metabolic, and inflammatory diseases, including obesity, type 2 diabetes, PD, AD, MS, depression, arthritis, and intestinal inflammation. Special attention has been paid to highlight and critically discuss current scientific evidence on the effects of phytochemicals on NLRP3 inflammasome pathways and their ability of counteracting central neuroinflammation, metabolic alterations, and immune/inflammatory responses in such diseases.

## 2. Mechanisms of NLRP3 Inflammasome Activation

NLRP3 inflammasome, the most characterized inflammasome sensor molecule, is a tripartite protein of the nucleotide-binding domain and leucine-rich repeat (NLR) family, containing an amino‑terminal pyrin domain (PYRIN) domain, a nucleotide-binding NACHT domain with ATPase activity, and a carboxy‑terminal leucine-rich repeat (LRR) domain [15]. NLRP3 is a key sensor of cellular stress, which senses changes in homeostatic cellular state. Currently, two modes of NLRP3 activation have been characterized: Canonical and non-canonical inflammasome activation. 

**Figure 1:**
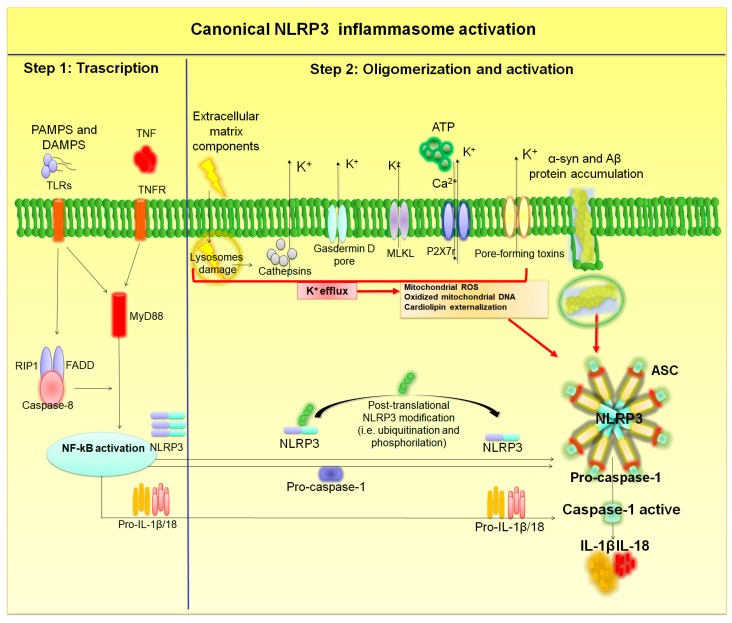
Diagram showing the different molecular mechanisms of canonical nucleotide-binding oligomerization domain leucine rich repeat and pyrin domain-containing protein 3 (NLRP3) inflammasome activation. The first step is regulated by toll-like receptors (TLRs)–adaptor molecule myeloid differentiation primary response 88 (MyD88) pathway and/or tumor necrosis factor receptor (TNFR), which activate pro-IL-1β and NLRP3 transcription via nuclear factor kB (NF-kB) activation. TLR4 stimulation by pathogen-associated molecular pattern molecules (PAMPs) and/or damage-associated molecular pattern molecules (DAMPs) can activate also the receptor-interacting protein 1 (RIP1)–FAS-associated death domain protein (FADD)-caspase-8 protein complex, which, in turn, promotes NF-kB transcription. The second step results in NLRP3 inflammasome oligomerization, leading to caspase-1 activation as well as IL-1β and IL-18 release. Permeabilization of cell membranes to potassium efflux (i.e., mixed lineage kinase domain-like protein (MLKL) activation, exposure to pore-forming Gasdermin D, P2X7 receptor activation by extracellular ATP, lysosomal damage, and cathepsin release) leads to a massive release of oxidized mitochondrial DNA, increase in mitochondrial reactive oxygen species (ROS) and cardiolipin externalization, which, in turn, promote NLRP3 inflammasome oligomerization and activation. α-Syn and Aβ protein accumulation, and post-translational NLRP3 modifications (i.e., phosphorylation and ubiquitination) can also promote the second step of NLRP3 inflammasome activation.

Canonical NLRP3 inflammasome activation requires two parallel and independent steps: Priming (transcription) and activation (oligomerization) (Figure 1) [16]. In the first step, innate immune signaling via toll-like receptor (TLR)-adaptor molecule myeloid differentiation primary response 88 (MyD88) and/or cytokine receptors, such as the tumor necrosis factor (TNF) receptor, promote pro-IL-1β and NLRP3 transcription via nuclear factor-κB (NF-κB) activation. In the second step, the oligomerization and activation of NLRP3 inflammasome lead to caspase-1 activation and, in turn, IL-1β and IL-18 processing and release [16,17]. Different stimuli, including viral RNA, inhibition of glycolytic or mitochondrial metabolism, extracellular osmolarity, α-synuclein (α-syn) and β-amyloid (Aβ) protein accumulation, degradation of extracellular matrix components, and post-translational NLRP3 modification (i.e., phosphorylation and ubiquitination) can initiate NLRP3 inflammasome oligomerization and activation. In addition, the permeabilization of cell membranes to potassium efflux (i.e., mixed lineage kinase domain-like protein (MLKL) activation, exposure to pore-forming gasdermin D, P2X7 purinergic receptor activation by extracellular adenosine triphosphate (ATP), lysosomal damage, and cathepsin release), the consequent release of oxidized mitochondrial DNA, the increase in mitochondrial reactive oxygen species (ROS), and the cardiolipin externalization can activate NLRP3 inflammasome assembly [1,10]. Independently from IL-1β maturation, caspase-1 activation promotes also pyroptosis, a key defense mechanism against microbial infections, which blocks the replication of intracellular pathogens, induces phagocytosis of surviving bacteria, and promotes the release of additional cytosolic proteins, such as high-mobility group box 1 (HMGB1) alarmin, a pro-inflammatory mediator significantly involved in the pathogenesis of several inflammatory chronic diseases [18,19].

**Figure 2:**
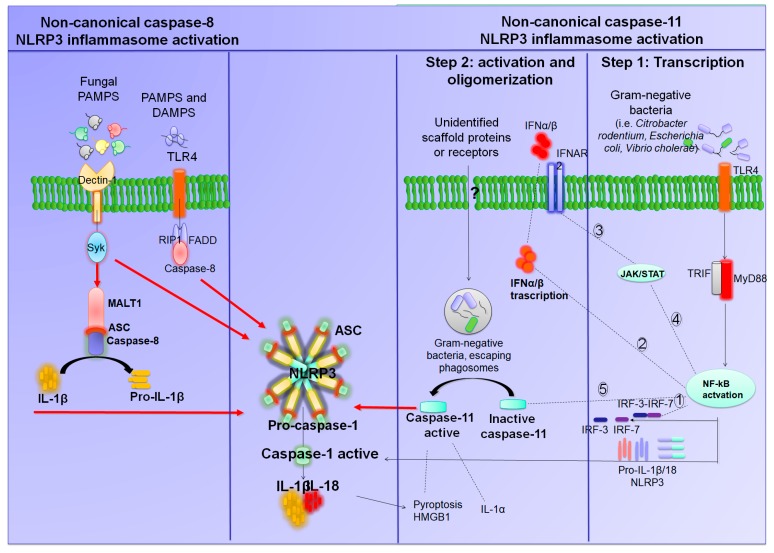
Diagram showing the different molecular mechanisms of non-canonical NLRP3 inflammasome activations. **Left panel**: Non-canonical caspase-8-dependent NLRP3 activation. TLR4 stimulation by PAMPs and/or DAMPs activates RIP1–FADD-caspase-8 intracellular signaling, which, besides promoting the NF-kB transcription step, can activate directly canonical NLRP3 oligomerization and assembly. In addition, fungi PAMPS (i.e., *Candida albicans*, fungal cell wall component β-glucans, and mycobacteria), via dectin-1 stimulation, can promote IL-1β transcription as well as the formation and activation of a mucosa-associated lymphoid tissue lymphoma translocation protein 1 (MALT1)–caspase-8– adaptor protein (ASC) complex, which contributes to processing and release of IL-1β. **Right panel**: Non-canonical caspase-11-dependent NLRP3 activation. In the first step, Gram-negative bacteria activate the TLR4–MyD88 and tumor necrosis factor receptor (TRIF) pathways, with consequent nuclear translocation of NF-κB, which promotes the transcription of IL-1β, IL-18, and NLRP3 as well as interferon regulatory factor (IRF)-3 and IRF7 genes. The IRF3–IRF7 complex (1) elicits the expression of IFN-α/β (2) that binds the interferon (IFN)-α/β receptor 1 IFNAR1/IFNAR2 receptor (3), leading to activation of the janus kinase/signal transducers and activators of transcription (JAK/STAT) pathway (4) and transcription of caspase-11 gene (5). In the second step, unidentified scaffold proteins or receptors induced by Gram-negative bacteria cleave and activate caspase-11, which induces pyroptosis as well as high-mobility group box 1 (HMGB1) and IL-1α release, and promotes the activation of NLRP3–ASC–caspase-1 pathway. Caspase-1 activation promotes also pyroptosis and HMGB1 release.

Besides canonical NLRP3 inflammasome activation, a non-canonical activation, which depends on caspase-11 in mice (caspase 4 and caspase 5 in humans), has been characterized (Figure 2) [1]. In this setting, in the first transcription step, gram-negative bacteria (i.e., *Citrobacter rodentium*, *Escherichia coli*, *Legionella pneumophila*, *Salmonella typhimurium*, and *Vibrio cholerae*) activate the TLR4–MyD88 and toll/IL-1 receptor homology-domain-containing adapter-inducing interferon-β (TRIF) pathways which, in turn, promote the transcription of IL-1β, IL-18, and NLRP3, as well as interferon regulatory factor (IRF)-3 and IRF7 genes via NF-κB activation. The IRF3–IRF7 complex promotes the expression of interferon (IFN)-α/β which, in turn, activates the IFN-α/β receptor 1 (IFNAR)/IFNAR2- the janus kinase/signal transducers and activators of transcription (JAK/STAT) pathway leading to transcription of caspase-11 gene. In the second step, unidentified scaffold proteins or receptors, induced by Gram-negative bacteria, cleave and activate caspase-11, which induces pyroptosis, HMGB1, and IL-1α release, and promotes IL-1β processing and release through activation of the canonical NLRP3-ASC-caspase-1 pathway (Figure 2) [1].

Of interest, an additional non-canonical caspase-8 dependent NLRP3 activation has been recently characterized (Figure 2) [20,21,22,23,24]. In particular, TLR4 stimulation by pathogen-associated molecular pattern molecules (PAMPs) and/or damage-associated molecular pattern molecules (DAMPs) can activate caspase-8 and its receptor-interacting protein 1 (RIP1)–fatty acid synthase (FAS)-associated death domain protein (FADD) protein, which, in turn, promote both the transcription step and canonical NLRP3 oligomerization and activation [24]. Moreover, fungi (i.e., *Candida albicans*), fungal cell wall component β-glucans, and mycobacteria, via dectin-1 stimulation, have been found to promote IL-1β transcription as well as the formation and activation of a mucosa-associated lymphoid tissue lymphoma translocation protein 1 (MALT1)–caspase-8–ASC complex that contributes to the processing and release of IL-1β (Figure 2) [22]. In particular, caspase-8 can act as a direct IL-1β-converting enzyme, instead of caspase-1. Indeed, IL-1β processing and caspase-8 activation were not evident in NLRP3^-/-^ or ASC^-/-^ bone marrow-derived dendritic cells (BMDCs) [20,23]. Moreover, the release of IL-1β from dendritic cells stimulated with a fungal infection occurred independently of caspase-1, through the association of ASC protein with caspase-8 [22]. 

Overall, this body of evidence points out that the activation of NLRP3 inflammasome is regulated by several molecular processes, which can be closely interconnected or occur independently. Nevertheless, further in vitro experiments on cultured cells are required to clarify the molecular mechanisms underlying the interplay between caspase-1, -8, and -11 in promoting the canonical and/or non-canonical NLRP3 activations.

## 3. Effects of Phytochemicals in NLRP3 Inflammasome-Related Diseases

**Figure 3:**
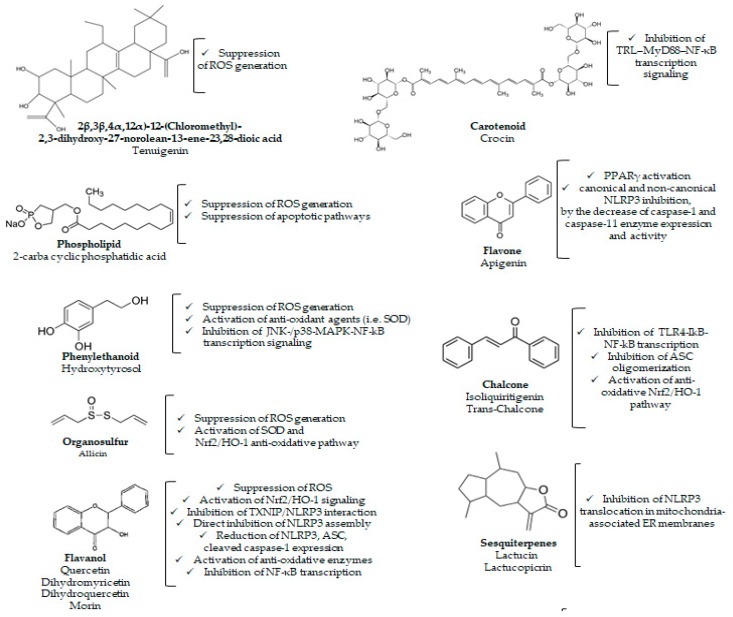

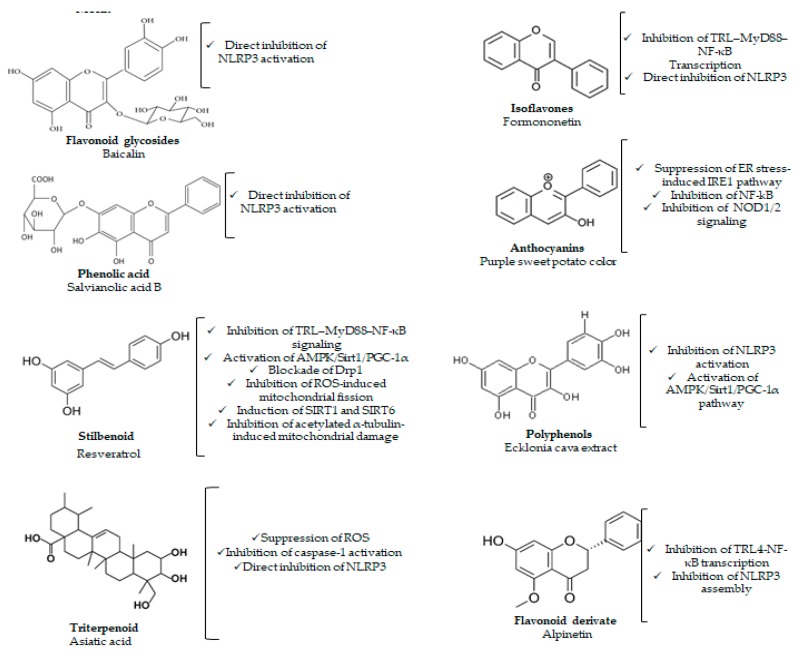

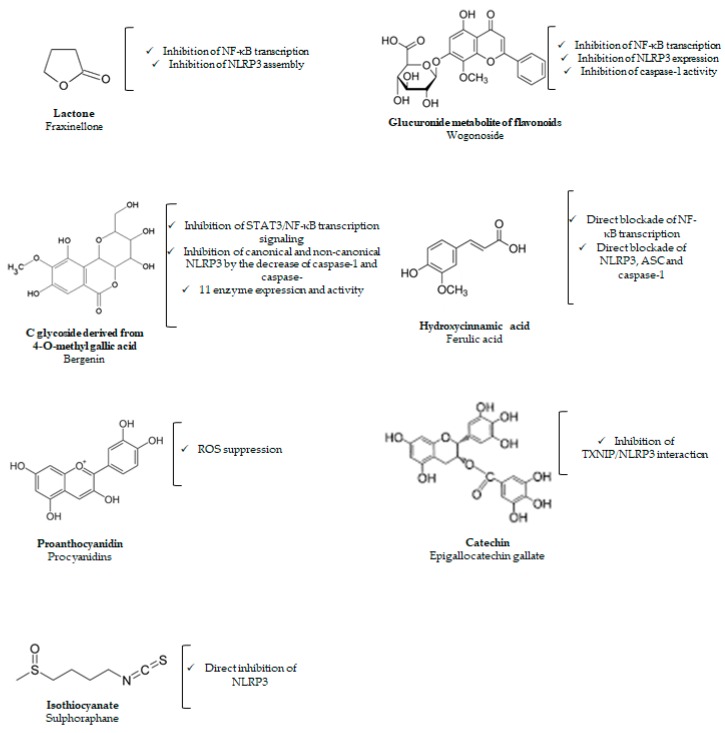
Chemical structures of different phytochemicals acting on NLRP3 inflammasome pathways and related molecular mechanisms through which these compounds modulate NLRP3 activation.

The involvement of inflammasome pathways in the pathophysiology of central nervous system (CNS) diseases, metabolic disorders, and chronic inflammatory diseases is fostering research on the potential therapeutic benefits resulting from the pharmacological targeting of NLRP3 inflammasome. In this field, besides specific programs of novel drug discovery, the scientific community is making intensive efforts to characterize the effects of natural compounds. Current evidence on the effects of different phytochemicals (see chemical structures in Figure 3) on NLRP3 inflammasome pathways and central neuroinflammation, metabolic alterations, and immune/inflammatory responses is addressed in the following sections and summarized in Table 1, Table 2 and Table 3.

### 3.1. Central Nervous System (CNS) Disorders

Increasing evidence supports the contention that central chronic neuroinflammation represents the main process implicated in the pathogenesis of several neurological and psychiatric disorders. Indeed, neuroinflammatory processes in the CNS have been documented in AD, PD, MS, ALS, and MDD patients at different stages of the disease. In this context, NLRP3 inflammasome is emerging as a pivotal driver in the onset of central neuroinflammation and consequent neurodegeneration [58,59], and several studies have shown an overactivation of NLRP3 inflammasome pathways in patients affected by these disorders. [6,60,61,62,63,64,65,66,67,68,69,70,71].

In support of this view, several studies in animal models of accelerated senescence, AD, PD, MS, ALS, and psychiatric disorders have shown a pivotal role of NLRP3 inflammasome in the onset and progression of central neuroinflammation and neurodegeneration [26]. Gordon et al. [6] observed an increase in IL-1β, caspase-1, NLRP3, and ASC expression in substantia nigra from PD mice induced by intranigral injection of 6-hydroxydopamine or α-syn preformed fibril, and that the pharmacological inhibition of inflammasome with MCC950, a recognized selective NLRP3 inhibitor, counteracted microglia activation, nigrostriatal degeneration, α-syn accumulation, and motor deficits in PD animals. Likewise, Zhang et al. [72] showed that mice subjected to chronic mild stress (a model of depression) displayed an increase in IL-1β, caspase-1, NLRP3, and ASC in hippocampus tissues, and that the pharmacological blockade of NLRP3 attenuated central and peripheral inflammation and ameliorated depressive-like symptoms. These results suggest that central NLRP3 overactivation shapes immune/inflammatory responses that contribute to neuroinflammation, microglial activation, neuronal loss, and cognitive and motor impairments in different CNS disorders, and that the pharmacological modulation of this enzymatic complex could represent a suitable way for treatment of such diseases. 

Of interest, in an attempt of understanding the role of NLRP3 inflammasome in the pathophysiology of CNS diseases, several efforts have been made to implement research on the effects of NLRP3 gene deletion and its components in preclinical models of brain disorders. A recent pioneering study by Haneka et al. [73] showed that NLRP3 gene deletion in amyloid precursor protein (APP)/presenilin 1 (PS1) mice (animal model of AD) attenuated learning and memory deficits, neocortex neuronal loss and brain Aβ accumulation, by increasing Aβ phagocytic clearance capacity and insulin-degrading enzyme (IDE) expression (an enzyme able to degrade extracellular Aβ). Bellezza et al. [74] reported that caspase-1 gene deletion in α-synA53T mice (a transgenic model of PD) counteracted central neuroinflammation and microglial activation [75]. These findings further support the view that NLRP3 inflammasome plays a key role in the pathophysiology of CNS disorders. In this regard, a recent and pioneering study by Venegas et al. [76] showed that central NLRP3 activation represent an early event in AD that contributes to Aβ deposition and disease progression. In particular, they observed that NLRP3 activation in the brain from AD mice promoted the release of ASC speck proteins in the extracellular space, which, in turn, rapidly bounded Aβ peptides, leading to an increase in its aggregation and accumulation [76]. These findings represent a point of novelty, since, for the first time, they demonstrate that, in AD, NLRP3 activation contributes to Aβ aggregation and accumulation. However, the molecular mechanisms underlying the interplay between Aβ and NLRP3 as well as its role in the pathophysiology of central neurodegeneration remain poorly understood. In addition, despite these interesting results, no distinction between canonical and/or non-canonical NLRP3 inflammasome activation in CNS diseases has been made. Indeed, at present, only a study by Martin et al. [77] has shown an involvement of caspase-8-dependent non-canonical NLRP3 inflammasome signaling in the pathophysiology of MS. In particular, the authors reported that the activation of non-canonical caspase-8-dependent inflammasome and the consequent massive release of IL-1β promoted Th17 cell differentiation and infiltration in brain tissues of experimental autoimmune encephalomyelitis (EAE) mice (a model of MS), thus contributing to diseases progression [77].

**Figure 4:**
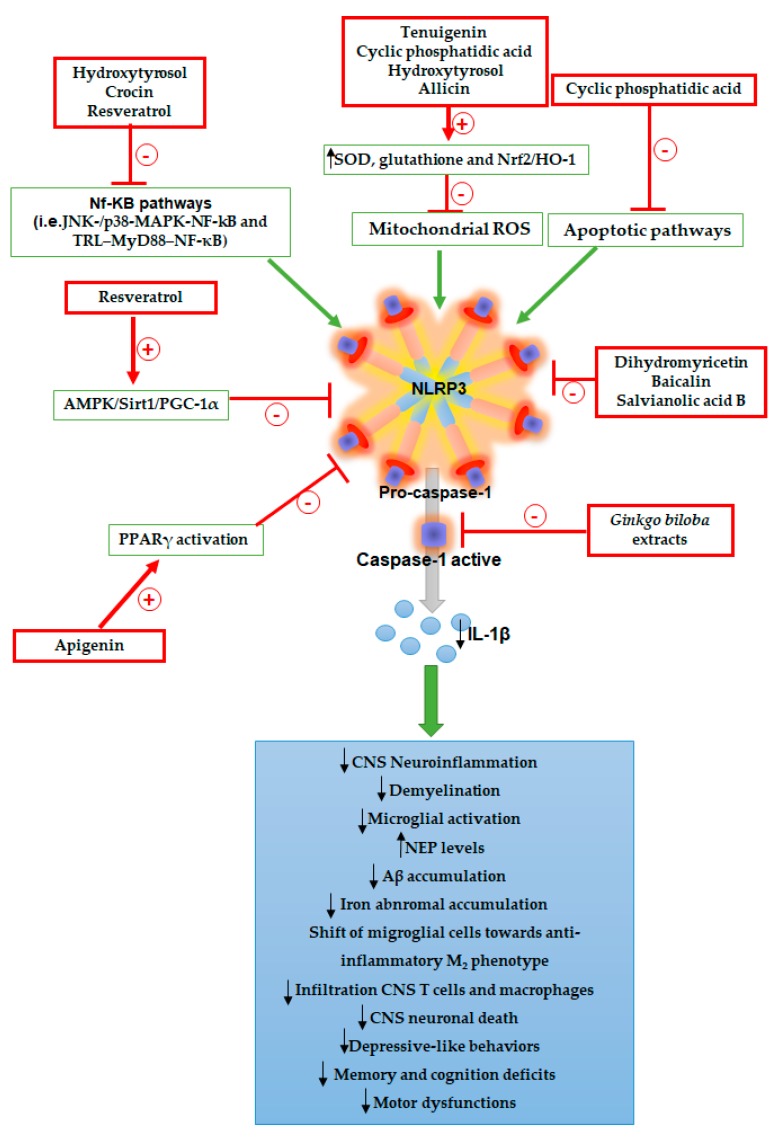
Diagram showing the molecular mechanisms through which phytochemicals can inhibit NLRP3 inflammasome signaling in the setting of CNS disorders.

Of interest, various phytochemicals have been found to inhibit NLRP3 activation acting on different steps of the inflammasome cascade and to exert beneficial effects in experimental models of CNS diseases, including PD, AD, MS, and psychiatric disorders (Table 1 and Figure 4). A pioneering study by Fan et al. [25] showed that tenuigenin, a natural extract from *Polygala tenuifolia* root, endowed with antioxidant, anti-aging, and anti-inflammatory properties, and able to cross the blood brain barrier (BBB), exerted beneficial effects in animals with MPTP-induced PD, through inhibition of NLRP3 activation and consequent decrease in IL-1β release [25]. The molecular mechanism underlying this effect was proposed to depend on the ability of tenuigenin to suppress ROS generation, thus suggesting that the blockade of NLRP3 upstream signaling in the CNS could represent a suitable therapeutic target for treatment of PD. Likewise, Yamamoto et al. [78] observed that in vivo treatment with cyclic phosphatidic acid (2ccPA), a natural phospholipid, counteracted demyelination, microglial activation, and motor dysfunctions in mice with cuprizone-induced MS, through NLRP3 inhibition via suppression of mitochondrial oxidative stress and apoptotic pathways. In addition, 2ccPA reduced the density of CD4+ T cells as well as macrophage infiltration in brain tissues from EAE mice. These results support the view that the inhibition of upstream NLRP3 signaling can also counteract the infiltration of CNS T cells and macrophages. Therefore, the blockade of NLRP3 activation in the CNS and the consequent decrease of immune/inflammatory cell infiltration could represent a suitable pharmacological target in the setting of CNS disorders.

Consistent with the above data, Peng et al. [27] observed that treatment with hydroxytyrosol (3,4-dihydroxyphenylethanol), a main polyphenol metabolite of oleuropein, attenuated neuronal impairment, central inflammation, and apoptotic activation in brain tissues from APP/PS1 mice, through the inhibition of NLRP3 inflammasome activation via suppression of ROS, known to activate NLRP3 inflammasome assembly [1]. In particular, hydroxytyrosol has been found to counteract ROS formation through the activation of antioxidant agents, including glutathione and superoxide dismutase. In addition, hydroxytyrosol decreased the expression of C-JunNH_2_-terminal kinase (JNK)-/p38-mitogen-activated protein kinase (MAPK)-NF-kB pathway, a molecular pathway that regulates the first step of NLRP3 activation. Therefore, the NLRP3 blockade by hydroxytyrosol could be dependent on ROS suppression and inhibition of JNK-/p38-MAPK-NF-kB transcription.

The inhibition of ROS generation as a suitable pharmacological target for inhibiting NLRP3 inflammasome assembly has been confirmed by a subsequent study, showing that in vivo administration of allicin, one of the main active compounds from garlic, attenuated depressive-like behaviors, CNS neuroinflammation, abnormal iron accumulation, and neuronal apoptosis in mice with chronic social defeat stress (CSDS), through NLRP3 inflammasome signaling inhibition by activation of antioxidant pathways and suppression of ROS generation. In particular, allicin increased SOD and nuclear factor (erythroid-derived 2)-like 2 (Nrf2)/heme oxygenase 1 (HO-1) anti-oxidative activities, that, in turn, suppressed ROS levels, thus inhibiting NLRP3 assembly and activation [28].

Besides upstream targeting the inflammasome pathway, a direct blockade of NLRP3 inflammasome assembly has been shown to exert anti-inflammatory effects in CNS disorders. Feng et al. [12] reported that dihydromyricetin, a flavonoid compound derived from the medicinal plant *Ampelopsis grossedentata* and able to cross the BBB, ameliorated memory and cognition deficits, increased neprilysin (NEP) levels (enzyme involved in Aβ clearance), reduced Aβ accumulation, and promoted the shift of migroglial cells towards the anti-inflammatory M_2_ phenotype in hippocampus and cortex in APP/PS1 mice, through direct inhibition of NLRP3 activation. In particular, dihydromyricetin decreased NLRP3, ASC, and cleaved caspase-1 expression in microglial cells from AD mice. These findings suggest that dihydromyricetin, by counteracting CNS neuroinflammation and Aβ accumulation, could represent a suitable therapeutic approach for the management of AD. Likewise, Liu et al. [30] observed that in vivo treatment of TgCRND8 mice (a transgenic model of AD) with *Ginkgo biloba* extracts improved cognitive functions, attenuated the loss of synaptic structural proteins, counteracted microglial activation, and decreased CNS neuroinflammation, through a direct blockade of caspase-1 activation. 

Consistent with the above data, two recent papers by Liu et al. [31] and Jiang et al. [32] reported that the direct inhibition of NLRP3 activation with two different phytochemicals attenuated CNS neuroinflammation in animal models of depression. These authors observed that baicalin (5, 6-dihydroxy-7-O-glucuronide flavonoid glycoside), a major polyphenol compound extracted from *Scutellaria radix* roots, and salvianolic acid B, a natural compound extracted from *Salvia miltiorrhiza*, counteracted depressive-like behaviors and central neurogenic/inflammatory responses in rats with chronic unpredictable mild stress (CUMS) and LPS-induced depression, respectively, via direct blockade of NLRP3 inflammasome assembly. Therefore, the direct inhibition of NLRP3 activation with phytochemicals might represent a suitable pharmacological strategy for treatment of CNS disorders.

Recent observations have shown that several polyphenols exerted beneficial effects on CNS disorders via inhibition of the primary TRL–MyD88–NF-κB step of NLRP3 inflammasome activation. Zhang et al. [33] showed that crocin, a carotenoid isolated from *Gardenia jasminoides* and *Crocus sativus*, counteracted CNS neuroinflammation, locomotor disability, and immobility time, in concomitance with a shift of M_1_ pro-inflammatory microglia towards M_2_ anti-inflammatory phenotype, in mice with LPS-induced depression, through the inhibition of NLRP3 transcription step. Qi et al. [34] and Liu et al. [79] reported that treatment with resveratrol (3, 5, 4′-trihydroxystilbene), a stilbenoid endowed with anti-aging, anti-inflammatory, antioxidant, and anti-apoptotic activities [35,80,81], improved cognitive and behavioral functions in mice with AD induced by intracerebroventricular injection of Aβ1-42 as well as in animals with ovariectomy-induced anxiety- and depression-like behaviors, by inhibiting the NF-κB/NLRP3 signaling. Resveratrol has been found to activate the anti-inflammatory 5′-adenosine monophosphate-activated protein kinase (AMPK)/sirtuin1 (Sirt1)/peroxisome proliferator-activated receptor-gamma coactivator-1alpha (PGC-1α) pathway. Such an effect could contribute further to the inhibition of NLRP3 activation. However, the authors did not demonstrate whether resveratrol influenced NF-κB/NLRP3/IL-1β and AMPK/Sirt1/PGC-1α pathways in independent ways, or whether the inhibition/activation of one can regulate the other. Thus, further investigations are needed to characterize the molecular mechanisms underlying the interplay between AMPK/Sirt1/PGC-1α and NF-κB/NLRP3/IL-1β pathways. 

A pioneering study by Li et al. [36] has shown that treatment with apigenin (4’,5,7-trihydroxyflavone), a bioflavonoid with anti-inflammatory and antioxidant activities, exerted beneficial effects in CUMS rats, by counteracting behavioral alterations, CNS inflammation, and oxidative stress, through the inhibition of NLRP3 inflammasome via activation of peroxisome proliferator-activated receptor gamma (PPARγ). Indeed, the concomitant administration of GW9662, a PPARγ antagonist, to CUMS rats counteracted the inhibitory effects of apigenin on NLRP3, thus suggesting that apigenin can exert antidepressant-like effects through the blockade of NLRP3 by activation of PPARγ. These results represent an interesting point of novelty, since they suggest that a dynamic interplay between NLRP3 signaling and PPARγ contributes to neurogenic/inflammatory responses in depression, and that PPARγ blockade could represent a suitable molecular target to modulate NLRP3 activation. 

### 3.2. Metabolic Disorders

Several lines of evidence have shown that obesity is characterized by a chronic low-grade systemic inflammation, that seems to contribute to the development of insulin resistance and the pathogenesis of type 2 diabetes mellitus (T2DM) [82,83]. Indeed, obese patients display an uncontrolled activation of innate immune/inflammatory cells, with consequent massive release of pro-inflammatory cytokines, which, in turn, interfere with several metabolic processes, including insulin synthesis and signaling, and blood glucose levels [83]. In this setting, the NLRP3 inflammasome complex has been found to act as a key immune sensor involved in shaping immune/inflammatory responses [7,84]. Clinical evidence has documented an increase in caspase-1 activity and IL-1β secretion in adipose tissue macrophages from obese and T2D patients, and these patterns were tightly correlated with a condition of insulin resistance [7,85,86,87]. However, the causal relationship of inflammasome activation with chronic inflammation, obesity, and T2D remains to be clarified. In this context, several research efforts have been made to investigate the effects of NLRP3 gene deletion and its components in pre-clinical models of obesity and diabetes. In particular, several studies have shown that NLRP3^-/-^, caspase-1^-/-^, and ASC^-/-^ mice were less susceptible to the development of obesity induced by high-fat diet (HFD). Others have reported that IL-1β gene depletion in HFD obese mice attenuated adipose tissue inflammation and insulin resistance [88,89,90].

Of interest, recent studies have shown that NLRP3 inflammasome activation in obese mice, besides sensing innate immune responses, also shapes adaptive immune responses through actions on accumulation and activation of T cells in adipose tissue, that, in turn, contribute to alter insulin sensitivity [7]. In particular, NLRP3 gene depletion in HFD obese mice significantly decreased the number of CD4+ and CD8+ effector memory T cells and increased naive T cells in adipose tissue [7,91,92].

At present, poor evidence is available about the beneficial effects of phytochemicals in animal models of metabolic disorders. 

In the setting of obesity, only one study has reported that treatment with isoliquiritigenin, a flavonoid with chalcone structure obtained by *Glycyrrhiza uralensis,* exerted beneficial effects in mice with HFD-induced obesity. Isoliquiritigenin attenuated body weight gain, insulin resistance, hyperglycemia, hypercholesterolemia, and adipose tissues inflammation, via inhibition of both steps of NLRP3 activation. Indeed, isoliquiritigenin inhibited both NF-kB transcription via TLR4/IkB signaling blockade and ASC oligomerization [13].

Two recent studies evaluated the effects of natural compounds acting on NLRP3 intracellular cascade in animal models of T2DM. In the first study, Shim et al. [37] observed that treatment with leaf extracts of *Cichorium intybus*, containing a variety of phytochemicals, such as lactucin and lactucopicrin sesquiterpene lactones, attenuated body weight gain, glucose metabolism, insulin resistance, as well as systemic and adipose tissue inflammation in mice with HFD-induced type-2 diabetes, via inhibition of NLRP3 activation. In addition, the leaf extracts of *Cichorium intybus* promoted the shift of M_1_ pro-inflammatory macrophages in adipose tissues towards the M_2_ anti-inflammatory phenotype, reducing the inducible nitric oxide synthase (iNOS) and TNF M_1_ markers and increasing the expression of Arg-1 and IL-10 M_2_ markers. The molecular mechanism was proposed to depend on the ability of lactucin and lactucopicrin of inhibiting the translocation of NLRP3 molecular components in mitochondria-associated endoplasmic reticulum (ER) membranes, a pivotal step in the process of NLRP3 activation [93,94]. In the second study, Li et al. [38] reported that treatment with resveratrol counteracted adipose tissue oxidative stress and inflammation in streptozotocin-induced diabetes through the blockade of NLRP3 activation. In particular, the inhibitory effects of resveratrol were ascribed to its ability to activate the anti-inflammatory AMPK system through the blockade of dynamin related protein-1(Drp1)- and ROS-induced mitochondrial fission and the consequent inhibition of thioredoxin-interacting protein (TXNIP)/NLRP3 interaction. These results, although generated in different animal models of metabolic disorders, show that natural compounds targeting both priming and activation steps of NLRP3 activation could represent suitable therapeutic options for the management of obesity and diabetes. 

Of interest, in the setting of metabolic syndromes, the majority of available studies have investigated the effects of NLRP3-targeting phytochemicals in complications associated with obesity and diabetes, including hepatic inflammation, non-alcoholic fatty liver disease (NAFLD), diabetic nephropathy, and cognitive impairment [29,39,40,41,42,43]. Wang et al. [39] observed that the dietary favonol quercetin alleviated hepatic oxidative stress, inflammation, and steatosis in rats with streptozocin-induced diabetes, through the blockade of TXNIP/NLRP3 interaction [39]. Likewise, Wang et al. [40] reported that purple sweet potato color (PSPC), containing anthocyanins, ameliorated the hepatic histopathologic damage associated with HFD-induced diabetes, through NLRP3 inflammasome inhibition. The molecular mechanisms were proposed to depend on the ability of PSPC anthocyanins of inhibiting ER stress-induced inositol-requiring enzyme 1 (IRE1) signaling as well as nucleotide-binding oligomerization domain-containing protein 1/2 (NOD1/2)-NF-kB transcription, thus suggesting that this class of flavonoids can act on both steps required for NLRP3 activation. In a subsequent paper, Yang et al. [41] showed that resveratrol counteracted hepatic inflammation in HFD mice, through the inhibition of NLRP3 assembly, by inducing the expression of Sirt1 and Sirt6 anti-inflammatory proteins. In particular, treatment with resveratrol decreased the levels of hepatic triglyceride along with TNF, IL-1β, and IL-6 pro-inflammatory cytokines. However, the authors did not investigate the molecular mechanisms underlying the interplay among SIRT1, SIRT6, and NLRP3 inflammasome. 

Two recent papers reported that phytochemicals acting on NLRP3 activation alleviated obesity-induced renal damage and diabetic nephropathy. In the first paper, Eo et al. [42] observed that *Ecklonia cava* polyphenol extract (ECPE) ameliorated the renal histopathological damage and inflammation in HFD-induced obese mice. In particular, treatment with ECPE decreased systemic and tissue parameters related to kidney inflammation, including body and kidney weight, sterol regulatory element-binding protein (SREBP-1), acetyl-coA carboxylase (ACC), as well as fatty acid synthase (FAS) and renal NFκB, MCP-1, TNF, and C-reactive protein expression, through the activation of the anti-inflammatory AMPK/SIRT1/PGC-1α pathway and the inhibition of NLRP3 activation. However, the authors did not clarify whether ECPE blocked directly the inflammasome assembly, or indirectly through the activation of anti-inflammatory pathways related to NLRP3 activation. In the second paper, Tao et al. [29] observed that the dihydroflavone dihydroquercetin exerted protective effects in rats with HFD/streptozotocin-induced diabetic nephropathy, by attenuating urine microalbumin excretion and renal histopathological lesions, through NLRP3 blockade via both inhibition of upstream NLRP3 signaling and acting directly on NLRP3 assembly. 

Besides the beneficial effects of phytochemicals targeting NLRP3 signaling on liver and kidney inflammation associated with diabetes, a recent study has shown that the isoflavone formononetin alleviated cognitive dysfunctions associated with diabetes via NLRP3 inhibition. In this setting, treatment with formononetin attenuated learning and memory deficiencies and decreased circulating and hippocampus levels of malondialdehyde (MDA), TNF, IL-1β, and IL-6 in mice with streptozocin-induced diabetes. The molecular mechanism underlying NLRP3 inflammasome blockade was proposed to depend on the ability of formononetin to block the priming TLR4/MyD88/NF-κB step involved in inflammasome activation through the inhibition of extracellular HMGB1. These findings represent a point of novelty, since they highlight a novel mechanism of inhibition of the first step of NLRP3 activation. [43].

### 3.3. Chronic Inflammatory Diseases 

An increasing number of phytochemicals, including phenols, polyphenols, triterpenoids, and isothiocyanates, have been found to exert beneficial effects in several animal models of chronic inflammatory diseases through the inhibition of NLRP3 inflammasome pathways.

#### 3.3.1. Inflammatory Bowel Diseases 

IBDs, including Crohn’s disease (CD) and ulcerative colitis (UC), comprise chronic and relapsing inflammatory disorders that affect the gastrointestinal tract [95]. Recent studies have shown that the NLRP3 inflammasome complex, besides acting as a key player in the maintenance of intestinal homeostasis, shapes innate immune responses during bowel inflammation, thus contributing to sustain the ongoing inflammatory processes, the disruption of enteric epithelial barrier through a deregulation of tight junction proteins (i.e., claudin-1, claudin-2, and junctional adhesion molecule-A), as well as epithelial cell apoptosis [1,96]. Recent clinical evidence has documented an increased IL-1β secretion from colonic tissues and macrophages of IBD patients, these patterns being correlated with the severity of the disease [8]. In addition, Liu et al. [97] showed an increased expression of NLRP3, ASC and IL-1β in the colonic mucosa from IBD patients, as compared with healthy controls.

In an attempt to better understand the role of NLRP3 inflammasome in the pathophysiology of bowel inflammation, several studies have investigated the effects of gene deletion and in vivo pharmacological modulation of NLRP3 inflammasome signaling in preclinical models of colitis [1]. In particular, it has been shown that the gene deletion of molecular components involved in both canonical and non-canonical NLRP3 inflammasome activation had both protective and detrimental roles in bowel inflammation, depending of the choice of experimental model. This led to postulate that, in the first phase of enteric inflammation, NLRP3 inflammasome contributes to tissue repair and maintenance of epithelial barrier integrity, while, in the chronic phase of inflammation, an overactivation of NLRP3 pathways leads to a massive release of IL-1β and IL-18, that contribute to immune/inflammatory responses and impairment of the intestinal epithelial barrier [1]. In support of this view, recent studies have shown that the in vivo pharmacological modulation of NLRP3 with drugs targeting different steps of NLRP3 activation, including inhibition of NF-kB transcription, protection against mitochondrial damage, activation of the Keap-1/NFE-related factor 2 (Nrf2) antioxidant pathway, inhibition of pro-caspase-1 cleavage, direct blockade of canonical and non-canonical NLRP3 activations, or IL-1β receptor exerted beneficial effects on bowel inflammation [98,99,100,101,102,103]. 

There is a large body of evidence that natural compounds targeting NLRP3 are able to exert anti-inflammatory effects on dextran sulphate sodium (DSS)-induced colitis in mice and colitis induced by 2,4,6-trinitrobenzenesulfonic acid (TNBS) in rats [1,11]. Guo et al. [44] observed that oral administration of asiatic acid, a natural triterpenoid compound, dose-dependently attenuated body weight loss, histological damage, myeloperoxidase activity, as well as colonic TNF, IL-1β, IL-6, and IFN- γ levels in mice with DSS-induced colitis through the inhibition of NLRP3 inflammasome activation. In particular, asiatic acid inhibited the upstream signaling of inflammasome activation by suppressing mitochondrial ROS generation, caspase-1 activation, and the inflammasome assembly [44]. 

The inhibition of NF-κB signaling and NLRP3 activation has been shown to also exert anti-inflammatory effects in colitis. Wu et al. [45] observed that treatment with fraxinellone, a natural lactone, reduced the weight loss, diarrhea, colonic macroscopic damage, enteric TNF, IL-1β, IL-6, and IL-18 levels, CD11b+ macrophage infiltration, as well as the mRNA levels of intercellular adhesion molecule 1 (ICAM1), vascular cell adhesion molecule 1 (VCAM1), iNOS, and cyclooxygenase-2 (COX-2) in mice with DSS-induced colitis, through NF-κB signaling and NLRP3 blockade. Likewise, treatment with wogonoside, a glucuronide metabolite of the bioactive flavonoid wogonin, exerted beneficial effects on bowel inflammation via direct inhibition of NF-κB and NLRP3 expression, as well as caspase-1 expression and activity [46]. These results indicate that phytochemicals targeting both steps of NLRP3 activation could represent a suitable and promising pharmacological target for treatment of bowel inflammation.

Consistent with the above data, He et al. [47] showed that the alpinetin, a flavonoid isolated from *Alpinia katsumadai Hayata*, attenuated diarrhea, colonic shortening, histological damage, and myeloperoxidase activity as well as colonic TNF and IL-1β expression in mice with DSS-induced colitis, likely by suppressing TRL4-NF-κB and NLRP3-ASC-caspase-1 signaling. However, the authors documented the ability of alpinetin of inhibiting NLRP3 activation in in vitro THP-1 cells, omitting the evaluation of alpinetin effects on NLRP3 activation in DSS mice. A recent study by Wu et al. [48] reported that formononetin alleviated the colonic shortening, histological damage, macrophage infiltration, colonic epithelial cell injury, and restored colonic tight junction (zonulin-1, claudin-1, and occludin) protein expression, by counteracting the increased expression of NLRP3 components (NLRP3, ASC, IL-1*β*) in DSS mice. These findings show, for the first time, that the isoflavone formononetin, via NLRP3 blockade, exerts beneficial effects on colitis both by counteracting colonic inflammation and restoring the integrity of epithelial barrier. 

Of interest, two pioneering papers by Marquez-Flores et al. [14] and Oficjalska et al. [49] have shown that the inhibition of canonical and non-canonical NLRP3 activation with polyphenols counteracted bowel inflammation in animals with DSS- and TNBS-induced colitis. In the first paper, Marquez-Flores et al. [14] observed that a dietary apigenin enrichment decreased the macroscopic and microscopic signs of colitis, and reduced colonic PGE, COX-2 and iNOS expression as well as serum matrix metalloproteinase (MMP-3) levels in DSS mice, through the inhibition of both canonical and non-canonical NLRP3 inflammasome pathways, through a decrease in caspase-1 and caspase-11 expression and activity. In the second study, treatment with bergenin, a polyphenolic compound, classified as a C glycoside derived from 4-O-methyl gallic acid, in rats with TNBS-induced colitis alleviated colonic macroscopic and microscopic damage, decreased neutrophilic infiltration, as well as colonic COX- 2 and iNOS expression and IL-1β, IFN-γ, and IL-10 levels, by modulation of STAT3 and NF-κB signaling and blockade of canonical and non-canonical NLRP3 inflammasome pathways. 

#### 3.3.2. Rheumatoid Arthritis

RA is a chronic autoinflammatory disease characterized by synovial inflammation and irreversible joint destruction, leading to joint functional impairment and often premature mortality [104]. Several lines of evidence support the view that NLRP3 inflammasome plays a pivotal role in the pathophysiology of RA [104]. Indeed, polymorphisms in different regions of NLRP3 gene have been associated with increased RA susceptibility and disease severity [105,106,107]. In addition, recent papers showed that RA patients are characterized by an increased mRNA and protein expression of inflammasome components, including NLRP3, ASC, caspase-1, and IL-1β levels in the synovia as well as circulating monocytes/macrophages, dendritic cells, and neutrophils, thus suggesting that the activation of NLRP3 inflammasome contributes both to tissues and systemic inflammation in RA [108,109,110,111,112]. However, current clinical evidence does not clarify whether NLRP3 inflammasome contributes to the pathophysiology of RA, or whether its activation occurs rather as a consequence of the initiation of synovial inflammatory processes. 

To better understand the pathophysiological role of NLRP3 in RA, several investigations have been performed in animal models of arthritis. Wei et al. [113] showed that IL-18^-/-^ mice were less susceptible to develop collagen-induced arthritis (CIA), as compared with wild-type animals, thus indicating a role of the pro-inflammatory cytokine IL-18 in the pathophysiology of RA. Guo et al. [9] observed an increased protein expression of NLRP3 and active caspase-1, along with an increment of IL-1β levels in the knee joint synovia and sera from CIA animals. In addition, they reported that the pharmacological blockade of NLRP3 with the selective inhibitor MCC950, through a decrease in the production of IL-1β, attenuated disease severity counteracting joint inflammation and bone destruction, thus suggesting that NLRP3-induced IL-1β release contributes to shaping and sustaining the immune/inflammatory processes in RA, and, most importantly, that inflammasome could represent a suitable pharmacological target for the management of RA [9].

However, there is very limited evidence that a natural compound targeting NLRP3 inflammasome can exert anti-inflammatory effects on experimental arthritis. Yang et al. [50] observed that oral administration of quercetin attenuated arthritic scores and paw edema decreased the joint levels of TNF, IL-6, PGE2, COX-2, iNOS, and Th17 cells and increased the number of Treg cells in mice with collagen-induced arthritis, through the inhibition of NLRP3 inflammasome activation. In particular, the authors found that quercetin inhibited the upstream signaling of NLRP3 activation via activation of anti-oxidant Nrf2/HO-1 signaling. As a confirmation, the application of HO-1 siRNA to fibroblast-like synoviocytes abolished the inhibitory effects of quercetin on NLRP3 activation [50]. 

#### 3.3.3. Gout

Gout is a chronic inflammatory arthritis characterized by an increase in urate concentrations and deposition of monosodium urate (MSU) crystals in joints [114,115]. In this context, increasing evidence supports the contention that NLRP3 inflammasome activation and the consequent massive release of IL-1β and IL-18 following MSU deposition promote mast cell, monocyte, and neutrophil influx into the synovium and joint fluids, thus contributing to the pathophysiology of gout [116,117,118]. In support of this view, several studies have shown that MSU crystals activated NLRP3 assembly in PBMCs from gout patients [119]. In addition, clinical trials have observed that the blockade of NLRP3 downstream signaling with IL-1 inhibitors, including rilonacept, canakinumab, and anakinra counteracted joint inflammation and attenuated the disease severity in patients with acute and chronic gout [120,121,122,123]. However, no clinical studies are available about the effects of direct blockade of IL-18 or its receptor in gout.

Of interest, the implementation of gouty arthritis animal models has allowed to better clarify the pathophysiological role of NLRP3 in gout. In particular, Nomura et al. [124] observed that NLRP3-/- mice were less susceptible to the development of MSU-induced inflammation [105]. Likewise, Martinon et al. [125] showed that MSU-induced gouty arthritis mice with gene deletion of inflammasome components, including caspase-1, ASC, and NLRP3, displayed a decrease in joint inflammation and neutrophil influx.

Several lines of evidence have shown that various NLRP3-targeting natural compounds can exert anti-inflammatory effects on MSU-induced gouty arthritis. Liu et al. [51] reported that administration of procyanidins, grape seed-derived natural flavonoids, attenuated the ankle circumference increase and joint inflammation in mice with acute MSU-induced gouty arthritis, through the inhibition of oxidative stress-induced upstream signaling of NLRP3 activation. Likewise, Jhang et al. [52] observed that epigallocatechin gallate, a bioactive polyphenol from green tea endowed with antioxidant activities, attenuated peritoneal inflammation in MSU animals via a blockade of TXNIP/NLRP3 interaction. In this study, epigallocatechin gallate decreased peritoneal neutrophil infiltration as well as the levels of neutrophil cytosolic factor 1, IL-6, IL-1β, monocyte chemoattractant protein-1 (MCP-1) in peritoneal lavage fluid, and amyloid A levels in serum, thus suggesting that NLRP3 blockade could represent a viable pharmacological strategy for the management of peritoneal inflammation associated with gout.

A pioneering study by Misawa et al. [53] showed that resveratrol administration to MSU mice exerted anti-inflammatory effects through the blockade of NLRP3 inflammasome assembly by reducing acetylated α-tubulin-induced mitochondrial damage. In particular, resveratrol, through the decrease in α-tubulin acetylation, prevented the optimal spatial conformation of NLRP3 and ASC, thus inhibiting inflammasome oligomerization and activation. These results show, for the first time, a novel mechanism of inhibition of upstream NLRP3 signaling by natural compounds.

Several studies have reported that different flavonoids exerted anti-inflammatory effects on experimental gout arthritis through the inhibition of both steps of NLRP3 activation. *Trans*-chalcone (1,3-diphenyl-2-propen-1-one), an open-chain flavonoid, attenuated knee joint inflammation and pain in MSU mice, via activation of antioxidative Nrf2/HO-1 pathway and inhibition of NF-κB/NLRP3 signaling [54]. Likewise, the administration of morin, a dietary bioflavonol, ameliorated ankle swelling, synovial hyperplasia, inflammatory cell infiltration, and cartilage degeneration as well as ankle levels of monocyte chemoattractant protein (MCP-1), iNOS, COX-2, and TNF, and IL-6 and IL-1β in rats with MSU-induced acute gouty arthritis, through the inhibition of NLRP3 activation by both increasing the activity of anti-oxidative enzymes, such as catalase (CAT) and SOD, and suppressing the NF-κB transcription step [55]. 

In further support of the above observations, a pioneering study by Doss et al. [56] showed that the intraperitoneal administration of ferulic acid, a phenolic phytochemical endowed with antioxidant, antihyperlipidemic, hypotensive, antimicrobial, anticarcinogenic, anti-inflammatory, and hepatoprotective activities, attenuated paw edema, elastase levels, lysosomal enzymes, inflammatory cell infiltration, and TNF and IL-1β levels in ankle joints in MSU rats. Such anti-inflammatory effects were ascribed to the ability of ferulic acid to inhibit directly both steps of NLRP3 activation. Indeed, the molecular docking analysis showed that ferulic acid displayed significant ligand efficiency towards pro-caspase-1, NF-κB, ASC, and NLRP3. These findings provide the first demonstration of direct molecular interactions between a phenolic phytochemical and NLRP3 inflammasome signaling, thus paving the way to the identification of novel phenolic compounds acting directly on NF-κB and NLRP3 components for the treatment of gouty arthritis.

One study has shown that sulforaphane (1-isothiocyanato-4-methylsulfinylbutane), a natural dietary isothiocyanate derivative, abundant in cruciferous vegetables, exerted anti-inflammatory effects on gouty arthritis via direct inhibition of NLRP3 inflammasome signaling [57]. Indeed, the oral administration of sulforaphane dose-dependently attenuated foot swelling and neutrophil recruitment while decreasing foot Il-1β levels and caspase-1 activity in animals with acute gout induced by MSU and air pouch. In vitro experiments indicated that sulforaphane suppressed MSU, ATP, and nigericin-induced NLRP3 inflammasome activation in bone-marrow-derived macrophages (BMDMs) [57], thus suggesting that sulforaphane could represent a promising phytochemical entity for prevention or treatment of gouty inflammation. 

## 4. Discussion

Current data from human studies suggest that NLRP3 inflammasome activation represents a common path to a variety of diseases, including neurological, psychiatric, metabolic, and chronic inflammatory disorders. Indeed, even though each disease displays distinct clinical, pathological, and genetic features, patients with PD, AD, MS, ALS, depression, obesity, diabetes, IBD, RA, and gout are characterized by an overactivation of the inflammasome signaling that contributes to central neuroinflammation, metabolic alterations, and immune/inflammatory responses in such disorders. However, human studies do not provide a clear causal relationship of NLRP3 activation with CNS, metabolic, or inflammatory diseases. 

The development of experimental models of neurological and psychiatric diseases, metabolic disorders, and chronic inflammatory diseases has allowed us to better understand the pathophysiological role of NLRP3 in these pathological conditions. Indeed, even though each experimental model displays distinct pathophysiological features, NLRP3 activation has been shown to contribute to central neuroinflammation, metabolic dysfunctions, and immune/inflammatory responses. In support of this view, gene depletion of inflammasome components or in vivo NLRP3 modulation with drugs acting at different levels of the inflammasome cascade in animal models have been found to counteract the progression of central neuroinflammation, metabolic alterations, and immune/inflammatory responses.

Based on the above considerations, and pooling together the available human and pre-clinical evidence, it is conceivable that the NLRP3 inflammasome complex represents a pivotal node for immune sensing in the innate immune system and that its activation in CNS, metabolic, and chronic inflammatory disorders contributes to shape the immune/inflammatory responses as well as to sustain the pathophysiological events underlying these diseases. In addition, NLRP3 activation has been shown to promote the adaptive immune responses, influencing the differentiation of T cells to pro-inflammatory Th17 phenotypes, thus suggesting that NLRP3 is not only one of the early immune sensors for innate immune response, but also for shaping of adaptive immune signals. Nevertheless, the exact role of NLRP3 in the pathophysiology of CNS, metabolic, and inflammatory disorders remains to be elucidated, with particular regard for the molecular mechanisms through which NLRP3 inflammasome can influence the adaptive immune system. Moreover, the majority of current human and pre-clinical studies have focused their attention on the role of canonical NLRP3 inflammasome activation, thus disregarding the evaluation of possible contributions by non-canonical caspase-8 and caspase-11-dependent NLRP3 activation pathways. This is a point of high interest, since an involvement of non-canonical caspase-8- and caspase-11-dependent inflammasome activation in the onset and progression of demyielinization and bowel inflammation, respectively, has been documented only in the setting of experimental MS and colitis. In addition, the role of other inflammasomes, including NLRP6, NLRP1, AIM2, and NLRC4, in the pathophysiology of CNS, metabolic, and inflammatory disorders remains unclear and poorly investigated [126,127,128]. Accordingly, there is a strong need for further studies aimed at characterizing the role of non-canonical NLRP3 inflammasome pathways and other inflammasomes in the pathophysiology of CNS, metabolic, and inflammatory diseases. 

Another crucial issue concerns the rick of adverse reactions following full inhibition of NLRP3, such as cancer observed in NLRP3-/- mice [129]. Several lines of evidence have shown that NLRP3-/- mice are more prone to develop colorectal cancer induced by azoxymethane/DSS, suggesting a protective role of NLRP3 inflammasome in tumorigenesis [130]. Conversely, others reported that NLRP3 inflammasome activation and the consequent release of IL-1β and IL-18 promoted tumor growth, proliferation, invasion, and metastasis in lung cancer, melanoma, breast cancer, and head and neck squamous cell carcinoma [129]. These findings suggest that NLRP3 inflammasome can play both protective and detrimental roles in tumors, likely depending on the tissue context. Therefore, further studies are needed to clarify the role of NLRP3 inflammasome in tumorigenesis, and to characterize the putative detrimental/protective effects associated with pharmacological agents acting as NLRP3 inflammasome inhibitors. 

Of note, despite several issues about the role of NLRP3 in the pathophysiology of CNS, metabolic, and inflammatory diseases that still need to be addressed, the beneficial effects resulting from NLRP3 modulation by phytochemicals in various experimental models allow us to postulate that the inhibition of NLRP3 with natural compounds could represent a viable pharmacological approach for the management of a variety of diseases. In this respect, current evidence shows that a number of polyphenols (i.e., flavonoids, stilbenoids, and phenols), triterpenoids, isothiocyanates, and carotenoids, acting on different steps of canonical or non-canonical NLRP3 inflammasome activation signaling, can counteract central neuroinflammation and neurodegeneration, metabolic alterations and related comorbidities, and immune/inflammatory responses in animal models of CNS, metabolic, and inflammatory diseases. However, in this area, a number of issues remain to be clarified: (1) What are the exact mechanisms through which 2ccPA and hydroxytyrosol inhibit NLRP3 activation in MS and AD, respectively? (2) What are the molecular mechanisms underlying NLRP3 inhibition by sulforaphane in gout? (3) How do some polyphenols block both canonical and non-canonical NLRP3 assembly? (4) Can phytochemicals targeting both canonical and non-canonical inflammasome activation be regarded as the most reliable pharmacological approaches for the management of CNS, metabolic, and inflammatory diseases? (5) Can a dietary supplementation with phytochemicals prevent or counteract neuroinflammatory and neurodegenerative processes, metabolic alterations, and immune/inflammatory responses? (6) Can phytochemicals exert beneficial effects in CNS, metabolic, and inflammatory diseases through the inhibition of other inflammasomes, including NRLP1, NLRP6, AIM2, and NLRC4? To clarify these points, intensive research efforts should be addressed to investigate, by means of molecular, biochemical, and pharmacological approaches, the effects of phytochemicals targeting NLRP3 inflammasome pathways and other inflammasomes in in vitro experiments on cultured cells and different experimental models.

## 5. Conclusions and Future Perspectives 

Current pre-clinical studies show that several phytochemical compounds can exert beneficial effects in CNS, metabolic, and inflammatory disorders through different modes of inhibition of NLRP3 inflammasome activation. In particular, polyphenols (i.e., flavonoids, stilbenoids, and phenols), triterpenoids, isothiocyanates, and carotenoids, acting on different steps of inflammasome activation, have been found to counteract central neuroinflammation and neurodegeneration, metabolic alterations, and immune/inflammatory responses in various experimental models. The main molecular mechanisms underlying NLRP3 blockade by phytochemicals have been ascribed to their ability of: (1) Inhibiting upstream NLRP3 activation by suppression of ROS generation; (2) directly blocking NF-kB-mediated transcription steps and/or NLRP3 oligomerization; (3) activating anti-inflammatory pathways, including AMPK/SIRT1/PGC-1α, that, in turn, could increase the expression of several ROS-detoxifying enzymes and inhibit directly NLRP3 assembly.

Based on this body of evidence, it can be proposed that phytochemicals acting at different steps of NLRP3 signaling could represent suitable pharmacological approaches for the management of a variety of diseases sharing the presence of chronic and persistent inflammatory conditions.

One considerable gap in our knowledge concerns whether these natural compounds can also interfere with non-canonical caspase-8 and/or caspase-11-dependent NLRP3 inflammasome activations. Another relevant issue stems from the lack of translational evidence about the effects of phytochemicals targeting NLRP3 in patients with CNS, metabolic, or inflammatory diseases. Despite some clinical trials that have shown the beneficial effects of phytochemicals in patients with CNS, metabolic, and inflammatory disorders, no evidence is currently available about the effects of natural compounds targeting NLRP3 in humans. In this respect, a translation of preclinical evidence into clinical practice could allow a better understanding of the protective effects of phytochemicals acting on NLRP3 in patients with CNS, metabolic, and inflammatory disorders. Unraveling these aspects could pave the way to novel therapeutic options for both the prevention and clinical management of neurological, psychiatric, metabolic, and inflammatory diseases.

## Figures and Tables

**Table 1:** Phytochemicals inhibiting nucleotide-binding oligomerization domain leucine rich repeat and pyrin domain-containing protein 3 (NLRP3) inflammasome activation in animal models of central nervous system (CNS) disorders.

Phytochemical	Category	Molecular Mechanisms	Dose and Treatment Time	Experimental Models	Ref.
**Tenuigenin**	***2β,3β,4α,12α)-12-(Chloromethyl)-2,3-dihydroxy-27-norolean-13-ene-23,28-dioic acid***	✓ Suppression of ROS generation	✓ 25 and 50 mg/Kg/day p.o. for 10 days	✓ MPTP-induced central dopaminergic neurodegeneration (toxin-induced model of PD)	[25]
**Cyclic phosphatidic acid**	***Phospholipid***	✓ Suppression of ROS generation ✓ Suppression apoptotic pathways	✓ 1.6 mg/Kg/day i.p. for 5 weeks	✓ Cuprizone-induced demyelination (animal model of MS) ✓ EAE mice (animal model of MS)	[26]
**Hydroxytyrosol**	***Phenylethanoid***	✓ Suppression of oxidative stress ✓ Activation of anti-oxidant agents (i.e., SOD) ✓ Inhibition of JNK-/p38-MAPK-NF-kB transcription signaling	✓ 5 mg/Kg/day p.o. for 42 weeks	✓ APP/PS1 mice (genetic model of AD)	[27]
**Allicin**	***Organosulfur***	✓ Suppression of ROS ✓ Activation of SOD and Nrf2/HO-1 anti-oxidative pathway	✓ 2, 10, 50 mg/Kg/day p.o. for 10 days	✓ CSDS mice (animal model of depression)	[28]
**Dihydromyricetin**	***Flavonolol***	✓ Reduction of NLRP3, ASC, cleaved caspase-1 expression	✓ 1 mg/Kg/day i.p. for 4 weeks	✓ APP/PS1 mice (genetic model of AD)	[29]
**Ginkgo biloba extracts**	***n.a***	✓ Direct blockade of caspase-1 activation	✓ 600 mg/day for 20 weeks	✓ TgCRND8 mice (a transgenic model of AD)	[30]
**Baicalin**	***Flavonoid glycosides***	✓ Direct inhibition of NLRP3 activation	✓ 20 and 40 mg/Kg7day p.o. for 3 weeks	✓ Chronic unpredictable mild stress (CUMS) rats	[31]
**Salvianolic acid B**	***Phenolic acid***	✓ Direct inhibition of NLRP3 activation	✓ 20 mg/Kg/day i.p. for 7 days	✓ LPS-induced depression rats	[32]
**Crocin**	***Carotenoid***	✓ Inhibition of TRL–MyD88–NF-κB transcription signaling	✓ 20 mg/Kg7day i.p. for 7 days	✓ LPS-induced depressive mice	[33]
**Resveratrol**	***Stilbenoid***	✓ Inhibition of TRL–MyD88–NF-κB transcription signaling	✓ 0.02 and 0.2 mg/Kg/day p.o. for 10 days	✓ Intracerebroventricular injection of Aβ1-42 (animal model of AD)	[34]
**Resveratrol**	***Stilbenoid***	✓ Inhibition of TRL–MyD88–NF-κB transcription signaling ✓ Activation of AMPK/Sirt1/PGC-1α pathway	✓ 20 mg/Kg/day i.p. for 14 days	✓ Ovariectomy-induced anxiety-and depression-like behaviors	[35]
**Apigenin**	***Flavone***	✓ PPARγ activation	✓ 3 mg/Kg/day p.o. for 3 weeks	✓ CUMS rats (animal model of depression)	[36]

**Table 2:** Phytochemicals inhibiting NLRP3 inflammasome activation in animal models of metabolic disorders and related comorbidities.

Phytochemical	Category	Molecular Mechanisms	Dose and Treatment Time	Experimental Models	Ref.
**Isoliquiritigenin**	***Chalcone***	✓ Inhibition of TLR4-IkB-NF-kB transcription ✓ Inhibition of ASC oligomerization	✓ 0.5% *w/w* for 20 weeks	✓ HFD-induced obesity	[13]
**Lactucin and** **Lactucopicrin**	***Sesquiterpene***	✓ Inhibition of NLRP3 translocation in mitochondria-associated ER membranes	✓ 50 mg/Kg/twice a week p.o. for 6 weeks	✓ HFD-induced type-2 diabetes	[37]
**Resveratrol**	***Stilbenoid***	✓ AMPK stimulation ✓ Blockade of Drp1 ✓ Inhibition of ROS-induced mitochondrial fission	✓ 50 mg/Kg7day p.o. for 7 days	✓ Streptozotocin-induced diabetes	[38]
**Quercetin**	***Flavonol***	✓ Inhibition of TXNIP/NLRP3 interaction	✓ 25 mg/Kg/day p.o. for 7 days	✓ Hepatic inflammation by streptozocin-induced type 1 diabetic rats	[39]
**Purple sweet potato color**	***Anthocyanins***	✓ Suppression of ER stress-induced IRE1 pathway ✓ Inhibition of NF-kB translocation ✓ Inhibition of NOD1/2 signaling	✓ 700 mg/Kg/day p.o. for 20 weeks	✓ Hepatic inflammation by HFD-induced diabetes	[40]
**Resveratrol**	***Stilbenoid***	✓ Induction of SIRT1 and SIRT6	✓ 8 mg/Kg/day p.o. for 4 weeks	✓ Hepatic inflammation associated with HFD-induced diabetes	[41]
***Ecklonia cava* extract**	***Polyphenol***	✓ Inhibition of NLRP3 activation ✓ Activation of AMPK/Sirt1/PGC-1α pathway	✓ 100 and 500 mg/Kg/day p.o. for 12 weeks	✓ HFD-induced diabetic nephropathy	[42]
**Dihydroquercetin**	***Dihydroflavone***	✓ Suppression of ROS ✓ Direct inhibition of NLRP3 assembly	✓ 25, 50, and 100 mg/Kg/day p.o. for 12 weeks	✓ HFD/streptozotocin-induced diabetic nephropathy	[29]
**Formononetin**	***Isoflavone***	✓ Inhibition of TRL–MyD88–NF-κB transcription signaling	✓ 25, 50 mg/Kg/day p.o. for 6 weeks	✓ Cognitive dysfunctions associated with streptozocin-induced diabetic	[43]

**Table 3:** Phytochemicals inhibiting NLRP3 inflammasome activation in animal models of chronic inflammatory diseases (colitis, arthritis, and gout).

Phytochemicals	Category	Molecular Mechanisms	Dose and Treatment Time	Experimental Models	Ref.
**Asiatic acid**	***Triterpenoid***	✓ Suppression of mitochondrial ROS ✓ Inhibition of caspase-1 activation ✓ Direct inhibition of NLRP3	✓ 3, 10, and 30 mg/Kg/days p.o. for 14 days	✓ DSS-induced colitis	[44]
**Fraxinellone**	***Lactone***	✓ Inhibition of NF-κB transcription ✓ Inhibition of NLRP3 assembly	✓ 7.5, 15, and 30 mg/Kg/day p.o. for 10 days	✓ DSS-induced colitis	[45]
**Wogonoside**	***Glucuronide metabolite of the bioactive flavonoid wogonin***	✓ Inhibition of NF-κB transcription ✓ Inhibition of NLRP3 expression ✓ Inhibition of caspase-1 activity	✓ 12.5, 25, and 50 mg/Kg/day p.o. for 10 days	✓ DSS-induced colitis	[46]
**Alpinetin**	***Flavonid derivate***	✓ Inhibition of TRL4-NF-κB transcription signalling ✓ Inhibition of NLRP3 assembly	✓ 25, 50, and 100 mg/Kg/day i.p. for 3 days before DSS treatment	✓ DSS-induced colitis	[47]
**Formononetin**	***Isoflavone***	✓ Direct inhibition of NLRP3	✓ 25, 50, and 100 mg/Kg/day i.p. for 7 days	✓ DSS-induced colitis	[48]
**Apigenin**	***Flavon***	✓ Inhibition of canonical and non-canonical NLRP3 inflammasome pathways, by the decrease of caspase-1 and caspase-11 enzyme expression and activity	✓ 3 g/day of diet resulting approximately to 125 mg/Kg/day for 30 days before DSS treatment	✓ DSS-induced colitis	[14]
**Bergenin**	***C glycoside derived from 4-O-methyl gallic acid***	✓ Inhibition of STAT3/NF-κB transcription signaling ✓ Inhibition of canonical and non-canonical NLRP3 activation by the decrease of caspase-1 and caspase-11 enzyme expression and activity	✓ 12, 25, and 50 mg/Kg/day p.o. for 3 days before the induction of colitis	✓ TNBS-induced colitis	[49]
**Quercetin**	***Flavonol***	✓ Activation of Nrf2/HO-1 signaling	✓ 150 mg/Kg/day p.o. for 14 days	✓ collagen-induced arthritis	[50]
**Procyanidins**	***Proanthocyanidin***	✓ Suppression of ROS	✓ 15, 30, and 60 mg/Kg p.o. 30 minutes before MSU treatment	✓ MSU-induced gouty arthritis	[51]
**Epigallocatechin gallate**	***Catechin***	✓ Inhibition of TXNIP/NLRP3 interaction	✓ 50 mg/Kg p.o. and s.c. before MSU treatment	✓ MSU-induced gouty arthritis	[52]
**Resveratrol**	***Stilbenoid***	✓ Inhibition of acetylated α-tubulin-induced mitochondrial damage	✓ 20 µg/Kg 60 min before MSU administration	✓ MSU-induced gouty arthritis	[53]
***Trans*-Chalcone**	***Chalcone***	✓ Activation of anti-oxidative Nrf2/HO-1 pathway ✓ Inhibition of NF-κB transcription signaling	✓ 30 mg/Kg p.o. 30 min before MSU administration	✓ MSU-induced gouty arthritis	[54]
**Morin**	***Flavonol***	✓ Activation of anti-oxidative enzymes (i.e., CAT and SOD) ✓ Inhibition of NF-κB transcription signaling	✓ 30 mg/Kg/day i.p. for 3 days	✓ MSU-induced acute gout arthritis	[55]
**Ferulic acid**	***Hydroxycinnamic acid***	✓ Direct blockade of NF-κB transcription signaling ✓ Direct blockade of NLRP3, ASC and caspase-1	✓ 30 mg/Kg i.p. 60 min before MSU administration	✓ MSU-induced acute gout arthritis	[56]
**Sulforaphane**	***Isothiocyanate***	✓ Direct inhibition of NLRP3	✓ 30 mg/Kg p.o. 60 min before MSU administration	✓ MSU-induced acute gout arthritis	[57]

## Abbreviations

α-syn	α-synuclein
Aβ	β amyloid
AD	Alzheimer’s disease
ALS	amyotrophic lateral sclerosis
AMPK	5′ adenosine monophosphate-activated protein kinase
AOM	azoxymethane
ASC	adaptor protein
ATP	adenosine triphosphate
CAPS	cryopyrin-associated autoinflammatory syndromes
CAT	catalase
DAMPs	damage-associated molecular pattern molecules
Drp-1	dynamin related protein-1
DSS	dextran sulphate sodium
ER	endoplasmic reticulum
FADD	FAS-associated death domain protein
HFD	high fat diet
IKb	IkappaB kinase
IRE1	Inositol-requiring enzyme 1
HMGB1	high mobility group box 1
HO-1	heme oxygenase 1
ip	intraperitoneal
IRF	interferon regulatory factor
IFN	interferon
IFNAR	interferon-α/β receptor
IL	interleukin
JAK/ STAT	janus kinase/signal transducers and activators of transcription
JNK	JunNH_2_-terminal kinase
MLKL	mixed lineage kinase domain-like protein
MALT1	mucosa-associated lymphoid tissue lymphoma translocation protein 1
MAPK	mitogen-activated protein kinase
MDD	major depressive disorder
MPTP	l-methyl-4-phenyl-l,2,3,6-tetrahydropyridine
MS	multiple sclerosis
MSU	monosodium urate
MyD88	myeloid differentiation primary response 88
*NF-kB*	nuclear factor kB
NLR	nucleotide-binding domain and leucine-rich repeat
NLRP3	nucleotide-binding oligomerization domain leucine rich repeat and pyrin domain-containing protein 3
NOD1/2	nucleotide-binding oligomerization domain-containing protein 1/2
NRF2/ARE	nuclear factor (erythroid-derived 2)-like 2/antioxidant response element
P2X	purinergic receptor 7
PAMPs	pathogen-associated molecular pattern molecules
PD	Parkinson’s disease
PGC-1α	peroxisome proliferator-activated receptor-gamma coactivator-1alpha
p.o.	oral
PPARγ	peroxisome proliferator-activated receptor gamma
PYRIN	amino‑terminal pyrin domain domain
RIP1	receptor-interacting protein 1
ROS	reactive oxygen species
s.c.	subcutaneous
Sirt1	sirtuin1
SOD	superoxide dismutase
STAT3	Signal transducer and activator of transcription 3
TLR	toll like receptor
TNBS	2,4,6-trinitrobenzenesulfonic acid
TNFR	tumor necrosis factor receptor
TRIF	toll/IL-1 receptor homology (TIR)-domain-containing adapter-inducing interferon-β
TXNIP	thioredoxin-interacting protein
